# Peptide Inhibitor of Complement C1 (PIC1) demonstrates antioxidant activity via single electron transport (SET) and hydrogen atom transfer (HAT)

**DOI:** 10.1371/journal.pone.0193931

**Published:** 2018-03-02

**Authors:** Magdielis Gregory Rivera, Pamela S. Hair, Kenji M. Cunnion, Neel K. Krishna

**Affiliations:** 1 Department of Microbiology and Molecular Cell Biology, Eastern Virginia Medical School, Norfolk, Virginia, United States of America; 2 Department of Pediatrics, Eastern Virginia Medical School, Norfolk, Virginia, United States of America; 3 Children's Specialty Group, Norfolk, Virginia, United States of America; 4 Children’s Hospital of The King’s Daughters, Norfolk, Virginia, United States of America; University of Kentucky, UNITED STATES

## Abstract

Reactive oxygen species (ROS) are natural byproducts of oxidative respiration that are toxic to organs and tissues. To mitigate ROS damage, organisms have evolved a variety of antioxidant systems to counteract these harmful molecules, however in certain pathological conditions these protective mechanisms can be overwhelmed. We have recently demonstrated that Peptide Inhibitor of Complement C1 (PIC1) mitigates peroxidase activity of the heme bearing proteins myeloperoxidase, hemoglobin, and myoglobin through a reversible process. To determine if this property of PIC1 was antioxidant in nature, we tested PIC1 in a number of well-established antioxidant assays. PIC1 showed dose-dependent antioxidant activity in a total antioxidant (TAC) assay, hydroxyl radical antioxidant capacity (HORAC) assay, oxygen radical antioxidant capacity (ORAC) assay as well as the thiobarbituric acid reactive substances (TBARS) assay to screen for PIC1 antioxidant activity in human plasma. The antioxidant activity of PIC1 in the TAC assay, as well as the HORAC/ORAC assay demonstrated that this peptide acts via the single electron transport (SET) and hydrogen atom transfer (HAT) mechanisms, respectively. Consistent with this mechanism of action, PIC1 did not show activity in a metal chelating activity (MCA) assay. PIC1 contains two vicinal cysteine residues and displayed similar antioxidant activity to the well characterized cysteine-containing tripeptide antioxidant molecule glutathione (GSH). Consistent with the role of the cysteine residues in the antioxidant activity of PIC1, oxidation of these residues significantly abrogated antioxidant activity. These results demonstrate that in addition to its described complement inhibiting activity, PIC1 displays in vitro antioxidant activity.

## Introduction

Oxidative stress is a natural byproduct of cellular metabolism. During this process, reactive oxygen species (ROS) or free radicals such as peroxides, superoxide, hydroxyl radical, and singlet oxygen are produced [1]. In addition to ROS, other non-radical reactive derivatives also known as oxidants (e.g., hydrogen peroxide, ozone, hypochlorous acid, lipid peroxide, etc.) are generated and can readily participate in free radical reactions [2]. Organisms have developed numerous enzymatic and non-enzymatic antioxidant systems to neutralize free radicals thus protecting tissue from oxidative damage. However, in situations of excessive amounts of oxidative stress during reperfusion injury or resulting from heme-based peroxidases, inflammation, ionizing radiation, or free metals (e.g., copper and iron), these antioxidant systems can become overwhelmed. Resultant ROS and oxidant formation that exceeds compensatory mechanisms damages cell structures by oxidizing proteins, nucleic acids and lipids [1]. Oxidative damage is associated with acute pathogenic processes including ischemia-reperfusion injury-mediated diseases [3] and hemoglobinemia-mediated acute kidney injury [4]. Oxidative tissue damage also contributes to a number of chronic diseases such as diabetes, atherosclerosis, arthritis, cancer and neurodegenerative diseases [5].

One of the most potent endogenous antioxidant molecules in the body is glutathione (GSH), a tripeptide which consists of a gamma peptide linkage between the carboxyl group of a glutamate side chain and the amine group of cysteine with the carboxyl group of cysteine attached by conventional peptide linkage to a glycine (reviewed in [6]). Glutathione functions as an antioxidant by virtue of its cysteine residue serving as an electron donor, upon which it is converted to its oxidized form, glutathione disulfide (GSSG). GSSG is reduced back to GSH by the enzyme glutathione reductase, using NADPH as an electron donor. Glutathione directly prevents ROS mediated damage to cellular components initiated by free radicals and heavy metals. Additionally, glutathione can collaborate with the thiol-dependent thioredoxin and glutathione antioxidant systems in cells to further inhibit oxidative stress. Finally, glutathione plays a critical role in activating the antioxidants vitamin C and E [6]. Underscoring the medical importance of glutathione, reduced glutathione, oxidized glutathione (GSSG), as well as total glutathione levels (GSH + GSSG) levels are routinely tested in clinical labs as a general assessment of a patient’s antioxidant reserves. Low levels of glutathione can be indicative of certain disease states in which ROS formation is consuming and overwhelming this critical compensatory mechanism [7].

Our laboratory has characterized a family of peptides known as Peptide Inhibitors of Complement C1 (PIC1) that inhibit the classical pathway of complement by binding the initiator molecule C1q and inhibiting activation of the cognate serine protease tetramer C1s-C1r-C1r-C1s that is normally associated with C1q [8,9]. We have recently demonstrated that the lead, pegylated PIC1 compound, PA-dPEG24 (IALILEPICCQERAA-dPEG24), dramatically inhibits the peroxidase activity of myeloperoxidase [10] as well as hemoglobin and myoglobin [11]. During these investigations we identified that PIC1 could reverse oxidized TMB to its non-oxidized state suggesting that PIC1 may be acting via an antioxidant mechanism. In this report, we explore the mechanisms by which PIC1 exerts antioxidant activity. We demonstrate that PIC1 possesses inherent antioxidant activity mediated by the vicinal residues Cys-9 and Cys-10 of the peptide and possesses an antioxidant activity similar to that of glutathione.

## Materials and methods

### Ethics statement

Human blood was obtained from healthy volunteers for the generation of pooled plasma used in these studies, per the Eastern Virginia Medical School IRB approved protocol 02-06-EX-0216. The study participants provided written informed consent.

### Materials

PIC1 (IALILEPICCQERAA-dPEG24) was manufactured by PolyPeptide Group (San Diego, CA) to ≥ 95% purity as verified by HPLC and mass spectrometry analysis. Ellman’s reagent (5,5’-dithio-bis(2-nitrobenzoic acid) and hydrogen peroxide (H_2_O_2_) were purchased from Thermo Fisher Scientific (Waltham, MA). Ascorbic acid, glutathione, ferrous chloride and ferrozine were purchased from Sigma-Aldrich (St. Louis, MO). OxiSelect™ Total Antioxidant Capacity (TAC) Assay Kit, OxiSelect™ Hydroxyl Radical Antioxidant Capacity (HORAC) Activity Assay and OxiSelect™ Oxygen Radical Antioxidant Capacity (ORAC) Activity Assay were purchased from Cell Biolabs, Inc. (San Diego, CA). The Thiobarbituric Acid Reactive Substances (TBARS) kit was purchased from ZeptoMetrix Corporation (Buffalo, NY).

### Total antioxidant capacity (TAC) assay

The TAC assay was performed using the OxiSelect™ Total Antioxidant Capacity Assay Kit as per manufacturer’s instructions (Cell Biolabs, Inc.). Briefly, increasing amounts of PIC1 or glutathione diluted in water were added to a 96 well microtiter plate. Titrating amounts of uric acid were also prepared in parallel to generate an antioxidant standard curve. Reaction buffer was then added and an initial absorbance recorded at 490 nm in a BioTEK plate reader. Next, copper ion reagent was added to each well, incubated for 5 minutes at room temperature followed by stop solution to terminate the reaction. The plate was then read again at 490 nm. To determine the total antioxidant capacity, mM uric acid equivalents extrapolated from the standard curve were converted to copper reducing equivalents as per the manufacturer’s instructions. Copper reducing equivalents are proportional to the sample’s total antioxidant capacity.

### Hydroxyl Radical Antioxidant Capacity (HORAC) assay

The HORAC assay was performed using the OxiSelect™ Hydroxyl Radical Antioxidant Capacity Activity Assay according to the manufacturer’s instructions (Cell Biolabs, Inc.). Briefly, increasing amounts of PIC1 or glutathione in assay diluent were added to a 96 well microtiter plate. Titrating amounts of gallic acid were also prepared in parallel to generate an antioxidant standard curve. Fluorescein solution was added to each well and incubated for 30 minutes at room temperature followed by the hydroxyl radical initiator and Fenton reagent. The plate was then immediately scanned in a BioTEK plate reader with an excitation wavelength of 480 nm and an emission wavelength of 530 nm at 5 minute increments for a total of 60 minutes.

### Oxygen Radical Antioxidant Capacity (ORAC) assay

The ORAC assay was performed using the OxiSelect™ Oxygen Radical Antioxidant Capacity Assay as per the manufacturer’s instructions (Cell Biolabs, Inc.). Briefly, increasing amounts of PIC1 or glutathione in assay diluent were added to a 96 well microtiter plate. Titrating amounts of Trolox^TM^ were also prepared in parallel to generate an antioxidant standard curve. Fluorescein solution was added to each well and incubated for 30 minutes at 37°C followed by the free radical initiator solution. The plate was then immediately scanned in a BioTEK plate reader at 37°C with an excitation wavelength of 480 nm and an emission wavelength of 520 nm at 5 minute increments for a total of 60 minutes.

### Thiobarbituric Acid Reactive Substances (TBARS) assay

The TBARS assay was performed using the Oxitek™ TBARS Assay Kit (ZeptoMetrix Corporation). Briefly, pooled human plasma was oxidized with 5 mM ferric chloride. Increasing amounts of PIC1 or glutathione was added to the oxidized plasma, incubated at 37°C for 30 minutes and then analyzed in the TBARS assay as per the manufacturer’s instructions. Supernatants were analyzed in a BioTek plate reader at 532 nm. Titrating amounts of malondialdehyde (MDA) were also prepared in parallel to generate a standard curve against which the plasma samples were plotted. The percent of oxidation inhibition was calculated using the formula % of inhibition = (OxPlasma–Sample/OxPlasma X 100.

### Metal chelating assay (MCA)

The MCA assay was performed as previously described [12] with some modifications. Briefly, 100 ul of PIC1 and glutathione at different concentrations in water were added to 500 ul of 0.2 mM ferrous chloride and 200 ul of 5 mM ferrozine solution. Ferrous chloride and ferrozine were dissolved in water. EDTA was used as an antioxidant control. Samples were incubated for 10 minutes at room temperature in the dark. Absorbance was then read at 562 nm in a BioTEK plate reader. The percent of MCA was calculated using the formula % MCA = Abs control–Abs sample/Abs Control X 100.

### Oxidation of PIC1

PIC1 (0.5mg) was added to increasing amounts of H_2_O_2_ in a microtiter plate, incubated for 15 minutes at room temperature followed by addition of Ellman’s reagent. Titrating amounts of cysteine were also prepared in parallel to generate a standard curve. The plate was then read at 412 nm in a BioTEK plate reader.

### Statistical analysis

Quantitative data were analyzed determining means, standard deviation, and Student’s t-test using Excel (Microsoft, CA). *P* values ≤ 0.05 were considered statistically significant.

## Results

### PIC1 displays antioxidant activity in a TAC assay

**Fig 1:**
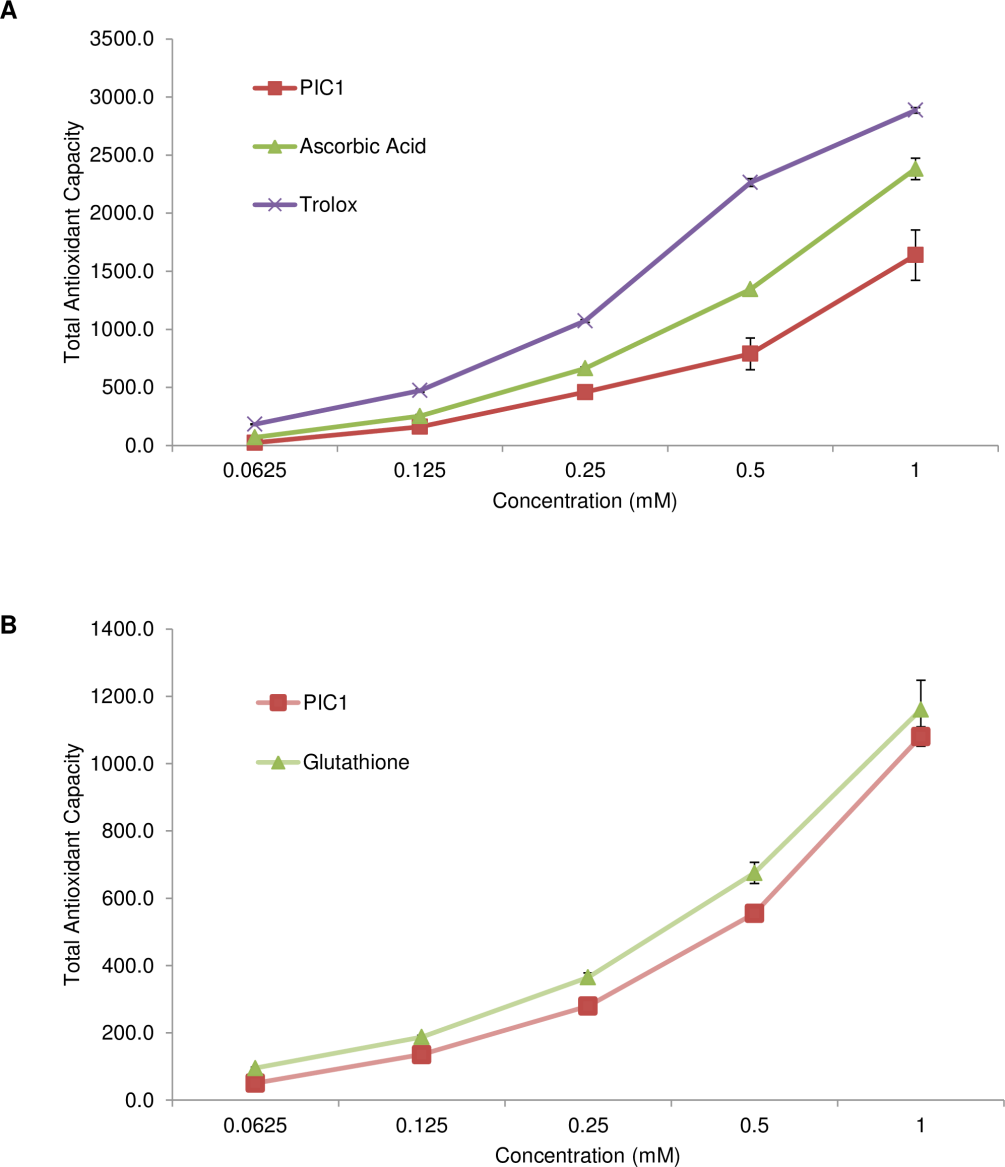
PIC1 displays antioxidant activity in the TAC assay. (A) Increasing amounts of PIC1 or the antioxidants ascorbic acid and Trolox^TM^ were incubated with reaction reagent in a microtiter plate. Titrating amounts of uric acid were also prepared in parallel to generate a standard curve. Data are means ± standard deviation of three replicate analyses. (B) Glutathione was analyzed in parallel with PIC1 in the TAC assay as described above. Data are means ± standard deviation of three replicate analyses.

To ascertain whether PIC1 displays antioxidant activity, a total antioxidant capacity (TAC) assay was utilized. This assay is used routinely to measure the total antioxidant capacity of molecules utilizing the single electron transport (SET) mechanism [13] which in this particular assay is demonstrated by the reduction of copper (II) to copper (I) by the antioxidant. Using uric acid as a standard curve, the total antioxidant power was calculated for PIC1. PIC1 displayed a dose-dependent antioxidant activity trend similar to the antioxidant molecules ascorbic acid and Trolox^TM^ (water soluble vitamin E) (Fig 1A). PIC1 did not reach the same level of antioxidant activity as these small antioxidant molecules. We then compared PIC1 to the archetypal antioxidant peptide, glutathione. PIC1 displayed similar dose response antioxidant activity to glutathione on a molar basis in this assay (Fig 1B). PIC1 showed increased total antioxidant capacity compared with glutathione at concentrations of 0.0625–0.5 mM (P < 0.02). Thus PIC1 displays antioxidant capacity via the SET mechanism that is equivalent or superior to glutathione.

### PIC1 shows antioxidant activity in the HORAC and ORAC assay

**Fig 2:**
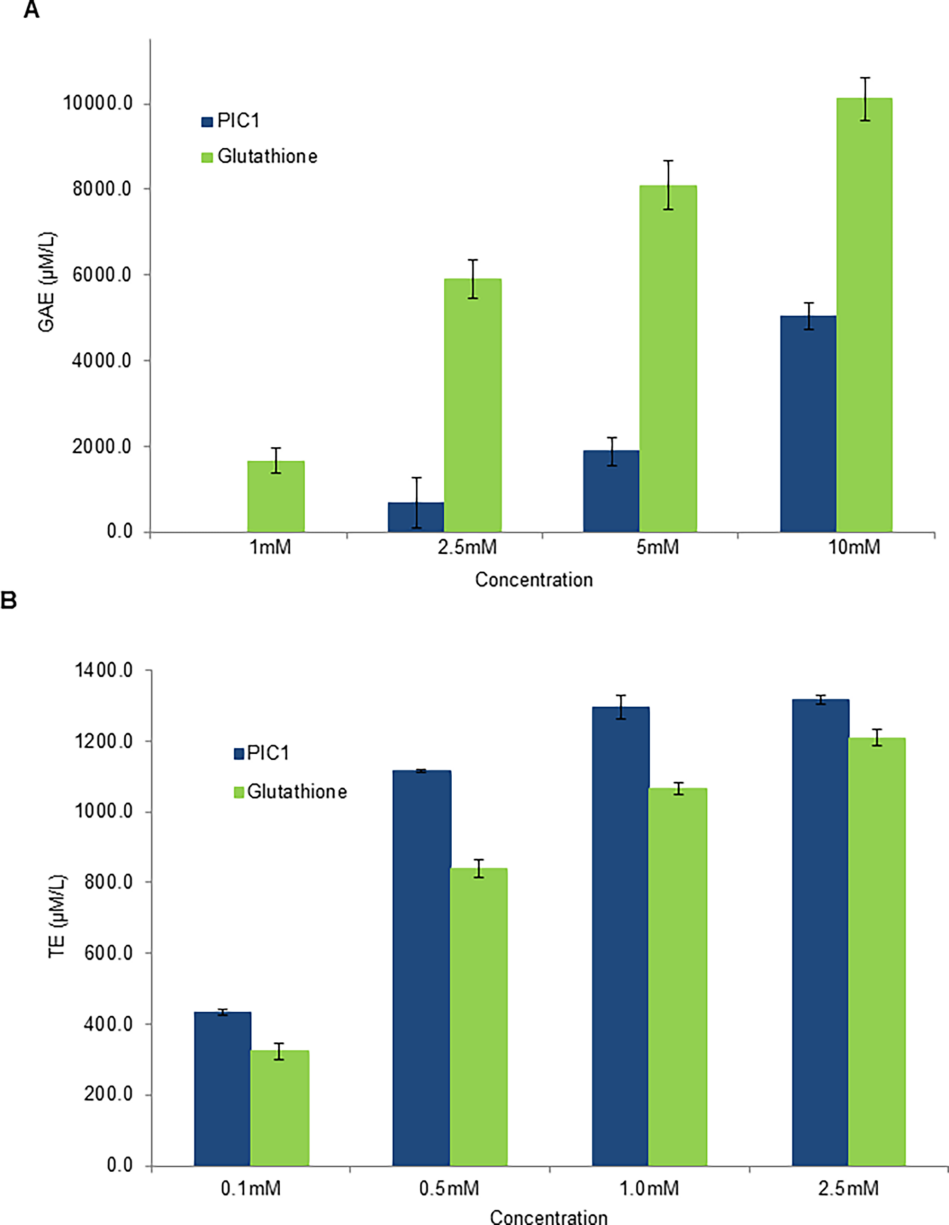
PIC1 displays antioxidant activity in the HORAC and ORAC assay. (A) For the HORAC assay, increasing amounts of PIC1 or glutathione were added to a microtiter plate. Titrating amounts of gallic acid were prepared in parallel to generate a standard curve. Data are means ± standard deviation of three replicate analyses. (B) The ORAC assay was performed in a similar manner to that of the HORAC assay except that a standard curve of Trolox^TM^ was utilized. Data are means ± standard deviation of three replicate analyses.

As PIC1 demonstrated a similar level of activity to glutathione in the TAC assay, we next tested whether PIC1 prevented ROS-induced oxidative stress in the HORAC and ORAC assays which are based on the oxidation of a fluorescent probe by either hydroxyl radicals or peroxyl radicals, respectively, via the hydrogen atom transfer (HAT) process [13]. For the HORAC assay, equimolar amounts of PIC1 and glutathione were evaluated against a standard curve of the antioxidant gallic acid. Both PIC1 and glutathione dose-dependently exhibited antioxidant activity with glutathione having a greater effect than PIC1 at concentrations of 2.5–10 mM (P<0.0003) (Fig 2A). When PIC1 and glutathione were evaluated in the ORAC assay using titrating amounts of Trolox^TM^ to generate a standard curve, both molecules displayed dose dependent antioxidant activity with PIC1 having a greater effect than glutathione at concentrations of 0.1–2.5 mM (P<0.002) (Fig 2B). These data suggest that compared to glutathione, PIC1 has less antioxidant activity against hydroxyl radicals and greater activity against peroxyl radicals.

### PIC1 demonstrates antioxidant activity in the TBARS assay

**Fig 3:**
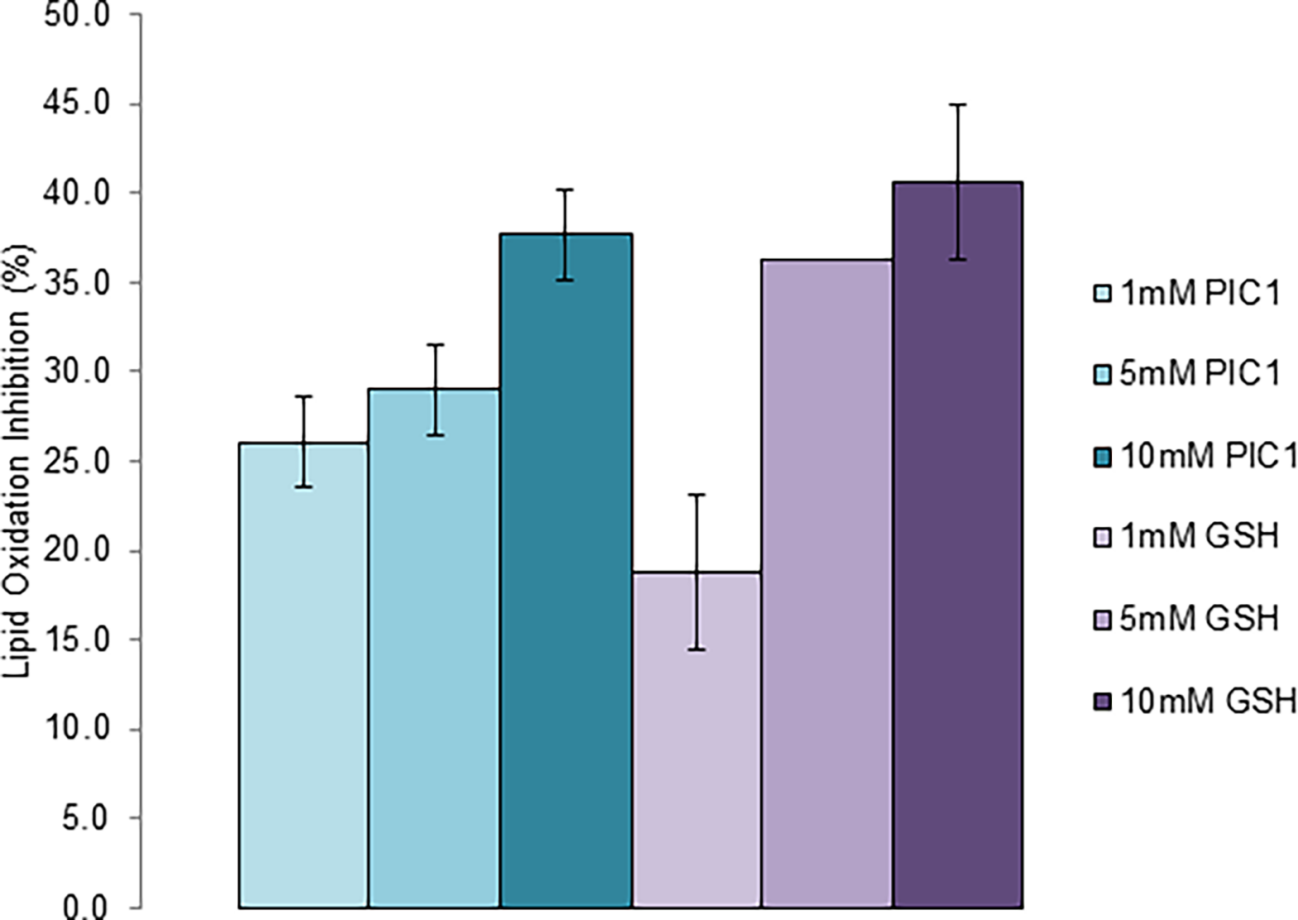
PIC1 shows inhibition of peroxidase activity in human plasma. Oxidized plasma was incubated with increasing amounts of PIC1 or glutathione and TBARS activity measured. Titrating amounts of MDA were prepared in parallel to generate a standard curve to plot the experimental samples. Data are means ± standard deviation of three replicate analyses.

Thiobarbituric acid reactive substances (TBARS), including lipid hydroperoxidases and aldelhydes, are found in biological specimens as a result of oxidative stress. Measuring their levels is routinely used to monitor peroxidation in human plasma or serum [14]. To assess whether PIC1 had antioxidant effects on peroxidation of human plasma, human plasma was oxidized with ferric chloride to generate TBARS. Increasing amounts of PIC1 or glutathione were added to the oxidized plasma, incubated at 37°C for 30 minutes and then analyzed for a reduction in TBARS levels. PIC1 and glutathione both dose-dependently inhibited peroxidase activity of oxidized plasma demonstrating no statistically significant difference at 1 mM or 10 mM concentrations (P > 0.05)(Fig 3). These results demonstrate the PIC1 has antioxidant activity against peroxidases present in human plasma.

### PIC1 does not show antioxidant activity in an MCA assay

**Fig 4:**
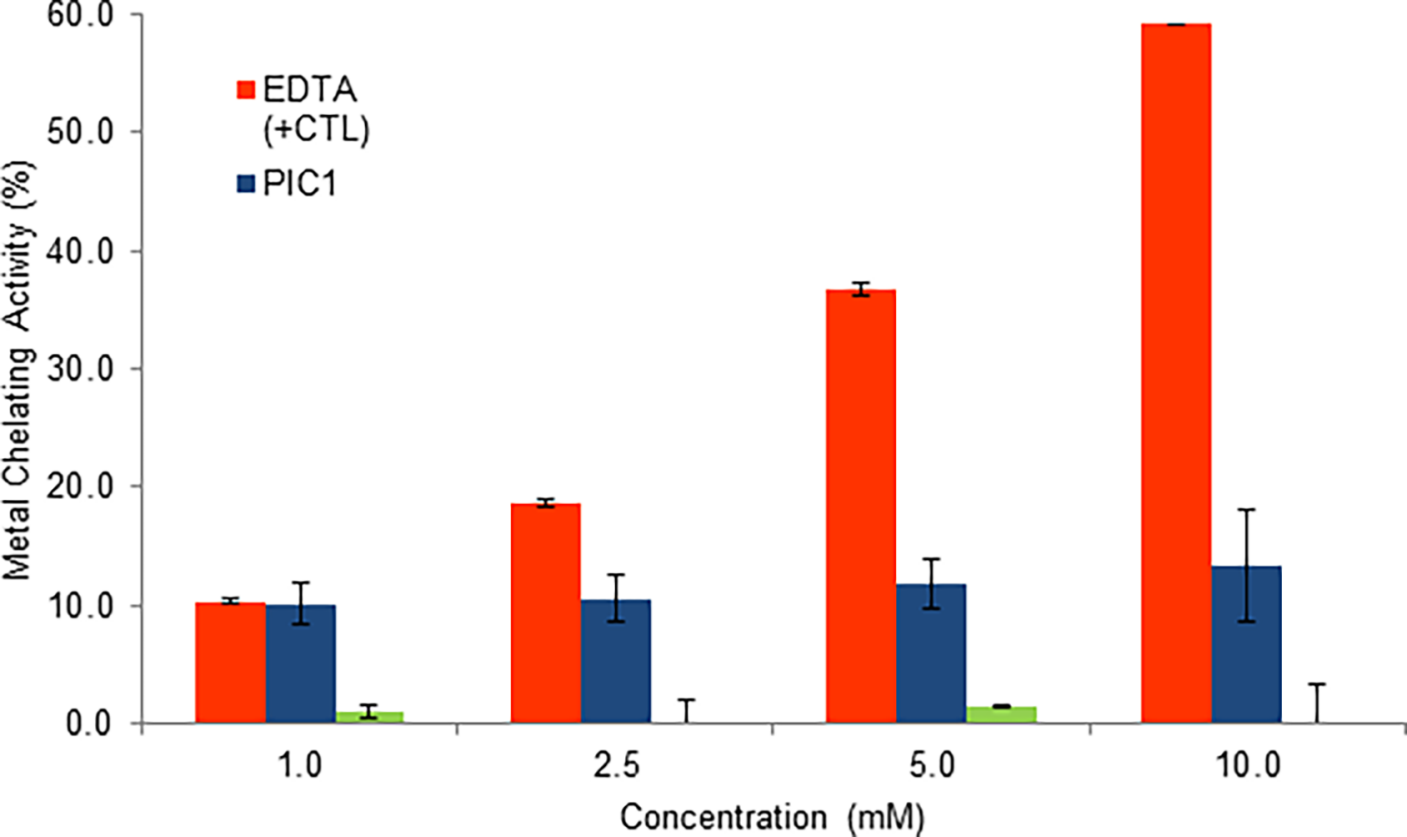
PIC1 shows no activity in the MCA assay. PIC1, glutathione and EDTA (positive control) at different concentrations were added to ferrous chloride and ferrozine solution for 10 minutes at room temperature. Absorbance was then read at 562 and the percent of MCA was calculated. Data are means ± standard deviation of three replicate analyses.

Certain antioxidants have been demonstrated to chelate metals preventing transfer of electrons and reducing free radical production. Metal chelating activity (MCA) can be demonstrated using ferrozine which can chelate with Fe^2+^ to form a red colored complex. Addition of a chelating antioxidant will limit reactivity of the ferrozine-Fe^2+^ complex resulting in a decrease of color that can be measured quantitatively at 562nm [12]. To ascertain whether PIC1 displays metal chelating antioxidant activity, this molecule along with glutathione was evaluated in the MCA assay. EDTA was included as a positive control for chelation and showed a dose dependent increase in metal chelating activity compared to PIC1 and glutathione neither of which demonstrated a dose response (Fig 4). These data suggest the antioxidant activity of PIC1 and glutathione is not mediated through a metal chelating mechanism, as expected.

### PIC1 antioxidant activity is mediated through its cysteine residues

**Fig 5:**
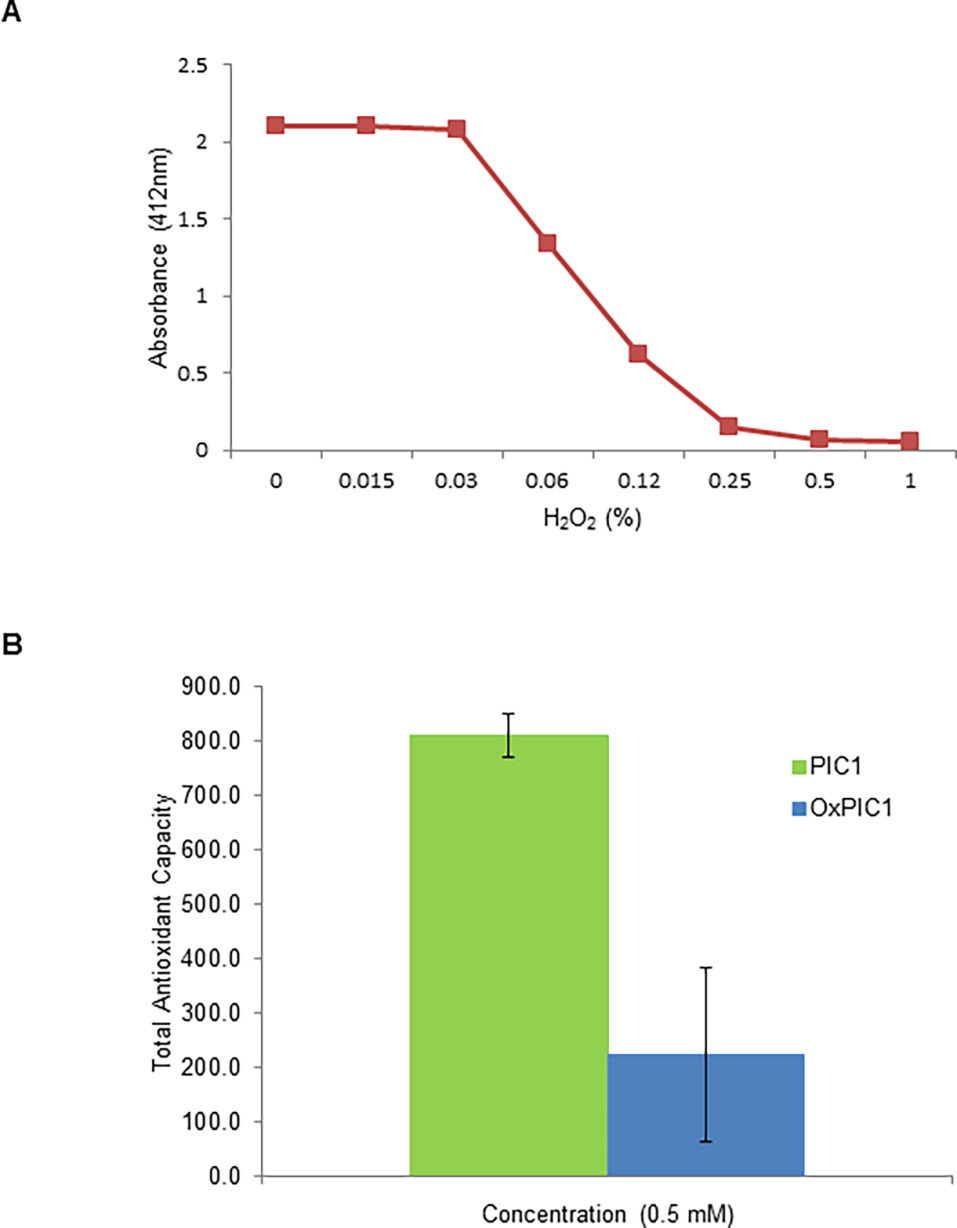
PIC1 antioxidant activity is mediated by cysteine residues. (A) PIC1 was added to increasing amounts of H_2_O_2_ in a microtiter plate and then incubated with Ellman’s reagent. (B) Reduced and oxidized PIC1 were incubated in a TAC assay. Titrating amounts of uric acid were also prepared in parallel to generate a standard curve. Data are means ± standard deviation of three replicate analyses.

In certain peptides and proteins the sulfhydryl group of the cysteine amino acid can manifest antioxidant activity by interacting with radical species by either hydrogen donation from the SH group or the loss of an electron from its sulfur atom [15]. The amino acid sequence of PA-dPEG24 is unique without significant homology with described naturally occurring proteins and peptides [8] and contains an unusual sequence structure with vicinal cysteine residues at positions 9 and 10. To determine whether the cysteine residues in this configuration contributed to the antioxidant activity of PIC1, we compared the antioxidant activity of PIC1 oxidized with H_2_O_2_ to reduced PIC1 in the TAC assay. To determine the concentration of H_2_O_2_ required to fully oxidize PIC1, the peptide was mixed with increasing amounts of H_2_O_2_ and then incubated with Ellman’s reagent, which reacts with reduced sulfhydryl groups producing a color change that can be detected at 412nm. Upon incubation of PIC1 with increasing amounts of H_2_O_2_, absorbance was reduced indicative of near complete oxidation of PIC1 at 0.5% H_2_O_2_ (Fig 5A). PIC1 treated with 0.5% H_2_O_2_ was then analyzed in the TAC assay along with reduced PIC1. Oxidized PIC1 had significantly less antioxidant activity compared to native PIC1 (P = 0.002) (Fig 5B). These data demonstrate that the vicinal cysteine residues of PIC1 play a major role in the antioxidant properties of the PIC1 molecule.

## Discussion

Previous work from our laboratory has demonstrated that PIC1 was able to reversibly inhibit the peroxidase activity of the heme-containing proteins myeloperoxidase [10], hemoglobin and myoglobin [11]. The observed behavior was consistent with an antioxidant mechanism of peroxidase inhibition and to test this hypothesis, we subjected PIC1 to a battery of well-established antioxidant assays. As a control in these experiments, we included the well characterized cysteine containing tri-peptide, glutathione. PIC1 demonstrated a similar antioxidant activity to glutathione in the TAC and ORAC assays (Figs 1 and 2B). In the HORAC assay, glutathione showed more antioxidant activity than PIC1 (Fig 2A) whereas PIC1 and glutathione displayed similar levels of peroxidase inhibition in human plasma using the TBARS assay (Fig 3). As expected, neither glutathione nor PIC1 demonstrated antioxidant activity in the MCA indicating that these peptides do not exhibit antioxidant activity via metal chelation (Fig 4). The antioxidant activity of PIC1 in TAC, HORAC and ORAC assays suggests that this peptide inhibits free radical formation via the HAT and SET mechanisms.

PIC1 derivative PA-dPEG24 harbors vicinal cysteine residues at positions 9 and 10 of the peptide. As cysteine is known to contribute significantly to the antioxidant activities of peptides [16,17], we assessed the involvement of the cysteine residues of PIC1 by treating this peptide with H_2_O_2_ and evaluating the function of the oxidized peptide in the TAC assay. Oxidized PIC1 had a diminished level of total antioxidant activity compared to reduced PIC1 demonstrating that the cysteine residues contribute significantly to the antioxidant activity of the peptide (Fig 5B). While other potent antioxidant peptides contain cysteine residues such as glutathione and the beta-lactoglobulin peptides (LTC and CQC) [16], to our knowledge there are only two other reports of an antioxidant protein and peptide that contain vicinal cysteine residues. Cysteine-3 and cysteine-4 of *Plasmodium falciparum* macrophage migration inhibitory factor have been demonstrated to possess antioxidant activity as well as a thioredoxin (Trx)-like oxidoreductase activity and these cysteine residues are essential for its activity [18]. A 6 amino acid peptide containing vicinal cysteine residues (I/L-N-I/L-C-C-N) isolated from the shortclub cuttlefish, *Sepia brevimana*, also has been demonstrated to have antioxidant properties [19]. Interestingly, this peptide has isoleucine and leucine residues at its N terminus as seen with PIC1. These non-polar residues have been reported to be a feature of some antioxidant peptides [17]. Additionally, PIC1 also contains a proline residue at position 7 which along with being a hydrogen donor may also contribute to the antioxidant properties of PIC1 by disrupting any secondary structure associated with the peptide thus positioning the cysteine residues in a desirable conformation for antioxidant activity [17].

Our data demonstrate that PIC1 possesses antioxidant activity in addition to its previously reported complement inhibiting activity [8,9]. PIC1 has shown efficacy in a pre-clinical model of hemolytic transfusion reaction where it inhibits complement-mediated destruction of transfused human red blood cells [20]. The ability of PIC1 to also block hemoglobin peroxidase activity from lysed human red blood cells [11] via an antioxidant effect as reported in this work suggests that this compound may be useful in further mitigating tissue damage in the pre-clinical scenario of hemolytic transfusion reaction as well as other inflammatory conditions in which reactive oxygen species are generated. Experiments to test this hypothesis are currently planned.

## Supporting information

S1 Fig PIC1 antioxidant activity in the TAC assay. (XLSX)

S2 Fig PIC1 antioxidant activity in the HORAC and ORAC assay. (XLSX)

S3 Fig PIC1 inhibition of peroxidase activity in human plasma. (XLSX)

S4 Fig PIC1 shows no activity in the MCA assay. (XLSX)

S5 Fig PIC1 antioxidant activity is mediated by cysteine residues. (XLSX)
